# Induction of mesenchymal stem cell chondrogenesis by polyacrylate substrates

**DOI:** 10.1016/j.actbio.2012.12.007

**Published:** 2013-04

**Authors:** Laurence Glennon-Alty, Rachel Williams, Simon Dixon, Patricia Murray

**Affiliations:** a Institute of Translational Medicine, University of Liverpool, Sherrington Building, Ashton Street, Liverpool L69 3GE, UK; b Institute of Ageing and Chronic Disease, University of Liverpool, Duncan Building, Daulby Street, Liverpool L69 3GN, UK; c Biomer Technology Ltd., Manor Park, Runcorn WA7 1SY, UK

**Keywords:** Mesenchymal stem cell, Cell culture, Chondrocyte, Polyacrylate, Surface chemistry

## Abstract

Mesenchymal stem cells (MSCs) can generate chondrocytes in vitro, but typically need to be cultured as aggregates in the presence of transforming growth factor beta (TGF-β), which makes scale-up difficult. Here we investigated if polyacrylate substrates modelled on the functional group composition and distribution of the Arg-Gly-Asp (RGD) integrin-binding site could induce MSCs to undergo chondrogenesis in the absence of exogenous TGF-β. Within a few days of culture on the biomimetic polyacrylates, both mouse and human MSCs, and a mesenchymal-like mouse-kidney-derived stem cell line, began to form multi-layered aggregates and started to express the chondrocyte-specific markers, Sox9, collagen II and aggrecan. Moreover, collagen II tended to be expressed in the centre of the aggregates, similarly to developing limb buds in vivo. Surface analysis of the substrates indicated that those with the highest surface amine content were most effective at promoting MSC chondrogenesis. These results highlight the importance of surface group functionality and the distribution of those groups in the design of substrates to induce MSC chondrogenesis.

## 1 Introduction

Over the years, many studies have explored the potential of different stem cell types, such as MSCs [1], to generate chondrocytes in vitro, the long-term aim being to determine if stem-cell-derived chondrocytes have the potential to repair osteoarthritic lesions [2,3]. In order to develop culture conditions to direct stem cells to differentiate to chondrocytes in vitro, it is important to understand the mechanisms that regulate the differentiation of these cells in vivo. Using this understanding will lead to the possibility of designing substrates that promote differentiation down this route.

One of the best studied models of chondrogenesis is the developing limb bud [4,5], where chondrocyte differentiation is initiated by the migration and subsequent condensation of mesenchymal cells to form tightly packed aggregates [4]. TGF-β signalling is important in these initial stages, its key role being to up-regulate the expression of the extracellular matrix (ECM) molecule, fibronectin, the cell adhesion molecule, N-cadherin, and the transcription factor, Sox9 [6–8]. Fibronectin is critical for the migration of the limb bud mesenchymal cells, and together with N-cadherin and Sox9, plays an important role in the aggregation process [9–11]. Functional analysis of the role of fibronectin in chondrogenesis has shown that the integrin-binding Arg-Gly-Asp (RGD) motif of fibronectin is critical for mesenchymal cell aggregation [11,12]. Mesenchymal cells within the aggregates then start to differentiate to become proliferating chondrocytes that express the chondrocyte-specific ECM proteins, collagen II and aggrecan [5]. Sox9 is crucial at this stage, being required to induce the genes that encode these ECM proteins [6,9,13]. As the limb bud matures, most of the chondrocytes in the developing long bones become hypertrophic, finally being replaced by bone tissue, and only chondrocytes at the ends of the long bones differentiate to hyaline cartilage. The mechanisms that control the differentiation of early proliferating chondrocytes to either hypertrophic or hyaline chondrocytes are not fully understood, but hypertrophic chondrocytes stop expressing Sox9 and begin to express mineralization markers [9], whereas articular hyaline chondrocytes continue to express Sox9 and collagen II [14].

Various types of stem cells, including MSCs [1], embryonic stem cells [15], amniotic-fluid-derived stem cells [16] and dermis isolated adult stem cells (DIAS cells) [17], have chondrogenic potential in vitro. However, most in vitro studies have focused on the regulation of MSC chondrogenesis. The standard method for inducing MSCs to undergo chondrogenesis in vitro involves growing the cells in micromass or pellet culture for several weeks in the presence of TGF-βs [1,18]; typically, TGF-β1, 2 or 3 are used to induce chondrogenesis, and BMP2, 4 or 6 are sometimes added as chondrogenic enhancers [19]. Under these conditions, MSC chondrogenesis resembles that which occurs in the developing limb bud; for instance, proliferating chondrocytes expressing Sox9 and collagen II start to differentiate centrally, and with time, some of these undergo further differentiation to become hypertrophic chondrocytes [20]. Peripheral cells within the MSC pellets express mineralization markers, such as alkaline phosphatase and osteocalcin, resembling the periosteal cells of the limb bud [20]. Although the standard method for MSC chondrogenesis is effective, the requirement for pellet culture and expensive growth factors makes scale-up problematic. Previous studies have shown that various types of biomaterial substrates can promote chondrogenesis without the need for pellet culture [21–28]. Nevertheless, in the aforementioned studies, there was still a requirement for TGF-β supplementation. More recently, matrix stiffness has been shown to have an impact on MSC differentiation; for instance, Park et al. have shown that in contrast to stiff substrates, soft substrates can induce MSC chondrogenesis to some extent, even in the absence of TGF-β, though the authors noted that adipocytes also differentiated under these conditions [29].

The aim of this study was to investigate if novel biomimetic polyacrylate substrates, designed to mimic the functional composition and distribution of the RGD integrin-binding site, were able to induce chondrogenesis of three stem cell types, namely mouse MSCs (mMSCs), mouse-kidney-derived stem cells (KSCs) and human MSCs (hMSCs) in the absence of exogenous growth factors. These substrates are complex multi-monomeric, acrylic based polymers, each monomeric unit containing specific functional groups, the composition and distribution of which can be discretely modified in terms of the starting monomer ratio in order to tailor the surface properties. The polymers are synthesized by free radical polymerization using proprietary controlled process techniques to ensure that the functional group chemistries of the constituent monomers are evenly distributed throughout the polymeric backbone. The composition and distribution of the functional group chemistries along the polymeric backbone influence the charge, charge density, hydrophobic/hydrophilic balance and surface stereochemistry of the resultant coating [30]. These substrates attempt to model the guanidinium and carboxyl groups, and their spatial distribution, using pendant amine and carboxyl or hydroxyl side groups distributed along a polyacrylate backbone.

## 2 Materials and methods

### 2.1 Substrate preparation

The following polyacrylates were fabricated using the Biomer Technology Ltd (BTL) proprietary polymerization technique: BTL15, ESP03, ESP04, ESP07. Each batch was tested for consistency of chain length by gel permeation chromatography. Polyacrylates differed in the proportion and distribution of amine, carboxyl and hydroxyl functional groups and the degree of steric hindrance present in the polymer chain, modulated by a ratio of ethyl to butyl side groups. All polyacrylate materials were supplied by BTL. Each of the polyacrylate-coated discs (Borosilicate Glass Co. UK) were prepared from the same w/w concentration of polymer in solvent using the same dip coating programme, at a thickness of ∼2 μm. The mechanical properties were not measured, but as the polyacrylates are all rigid glassy materials, their mechanical properties are expected to be similar. Substrates were sterilized prior to cell culture with ultraviolet light (265 nm).

### 2.2 Dynamic contact angle (DCA)

DCA was recorded using the Willhelmy method with a Cahn DCA322 microbalance and analysis software WinDCA32 (Thermo Cahn, USA). Advancing and receding contact angle was calculated for each sample in distilled water (72.6 dyne cm^–1^).

### 2.3 X-ray photoelectron spectroscopy (XPS)

XPS was conducted using an NCESS ESCA300 XPS spectrometer (VG Scienta). Survey, valence, C1s, O1s and N1s spectra were analysed at take-off angles of 45° and 15° at 150 eV, 0.8 mm slit, 1.8 kW. To prevent the samples from charging due to emission of photoelectrons, the sample surface was bombarded with a low-energy electron flood gun (Scienta FG300) within the analysis chamber, with the gun settings adjusted for optimum spectral resolution. Spectra curves were analysed by curve fitting, conducted using CasaXPS (Casa software Ltd) and OriginPro 7.5 SR6 (OriginLab Corporation, MA, USA).

### 2.4 Surface amine assay

The relative amine content of substrates was measured using sulfo-NHS-Biotin (Thermo) to bind to surface amines, followed by streptavidin-horse radish peroxidase (Bioscience) to generate a colorimetric enzyme assay with 3,3′,5,5′-tetramethylbenzidine (TMB) (Sigma). Sulfo-NHS-Biotin is typically used to biotinylate proteins and antibodies for detection, immobilization or purification [31,32]. Substrates were incubated with a 1 mg ml^−1^ solution of sulfo-NHS-biotin in PBS for 1 h, washed once in 50 mM glycine in PBS, and after washing with PBS, were incubated in 10 mM Streptavidin-HRP (Sigma) in PBS for 30 min, followed by three washes in PBS. Substrates were then incubated with TMB solution (Sigma) for 15 min. The reaction was halted by adding 1 N HCl (Sigma) and the optical density read at 450 nm. Specificity was confirmed by blocking with NHS-acetate (Thermo), under the same conditions.

### 2.5 Cell culture

For routine culture, D1 mMSCs (ATCC®) and H6 mKSCs [33] were maintained in MSC medium (Dulbecco’s modified Eagle medium (DMEM, Invitrogen), 10% FCS (PAA), 2 mM L-glutamine (Invitrogen)). Primary hMSCs (Lonza Walkersville Inc.) were cultured using MSCGM™ BulletKit® (Lonza), according to the manufacturer’s guidelines_._ For culture on polyacrylate substrates, mMSCs, mKSCs and hMSCs were seeded onto 15 mm diameter glass discs (polyacrylate-coated or uncoated controls) in 24-well plates (Nunc) in 250 μl droplets containing 1 × 10^4^ cells (mMSC and mKSC) or 5 × 10^3^ cells (hMSC). Medium was topped up to 0.5 ml after 1 day, then replaced every 3 days. For MSC chondrogenesis in standard pellet culture, a protocol adapted from Peister et al. was used [34]. In brief, 2 × 10^5^ mMSCs in 0.5 ml 10% (v/v) FCS were transferred to a 15 ml falcon tube and pelleted by centrifugation. After 1 day, medium was replaced with chondrogenic medium (high-glucose DMEM supplemented with 10 ng ml^−1^ TGFβ3, 500 ng ml^−1^ BMP-6 (Sigma), 0.1 μM dexamethasone (Sigma), 50 μg ml^−1^ ascorbate-2-phosphate (Sigma), 40 μg ml^−1^ proline (Invitrogen), 100 μg ml^−1^ pyruvate (Invitrogen), 50 mg ml^−1^ ITS + 3 liquid supplement (Invitrogen)). hMSCs were cultured as above, except that they were grown in hMSC chondro bulletkit (Lonza) supplemented with 10 ng ml^−1^ TGFβ3, according to the manufacturer’s guidelines.

### 2.6 Immunofluorescence

Cells were fixed using 4% (w/v) paraformaldehyde, and immunofluorescence and microscopy were performed as previously described [35]. The primary antibodies used were as follows: anti-mouse/human type II collagen antibody (CIICI; Hybridoma bank, NIH), anti-mouse osteocalcin (OG1; Santa Cruz) and anti-human osteocalcin (Osteocalcin; Santa Cruz).

### 2.7 Quantitative reverse transcriptase PCR (qPCR) analysis

Total RNA was extracted using Trizol® reagent (Invitrogen) according to the manufacturer’s instructions. All RNA was treated with RQ1 DNase (Promega) and reverse transcribed using Superscript III (Invitrogen) primed with 200 ng μl^−1^ random hexamers (Abgene). qPCR was conducted on a Corbett Rota Gene RG-300 using SYBR® Green Jumpstart™ Taq ReadyMix™ (Sigma) and 0.25 μM forward and reverse primers. Expression of each target gene was normalized to GAPDH and presented relative to expression on glass substrate controls. Primer sequences were as follows: mGAPDH F-TGAAGCAGGCATCTGAGGG, R-CGAAGGTGGAAGAGTGGGAG; mCol2a1 F-CTGACCTGACCTGATGATACC, R-CACCAGATAGTTCCTGTCTCC; mAcan F-CTCAGTGGCTTTCCTTCTGG, R-CTGCTCCCAGTCTCAACTCC; mBglap F-GACCATCTTTCTGCTCACTC, R-TCACTACCTTATTGCCCTCC; mCdh2 F-CCGTGAATGGGCAGATCACT, R-TAGGCGGGATTCCATTGTCA; Sox9 F-TACGACTGGACGCTGGTGCC, R-CCGTTCTTCACCGACTTCCTCC; mWt1 F-CCAGTGTAAAACTTGTCAGCGA, R-TGGGATGCTGGACTGTCT; hGAPDH F-GTGGTCTCCTCTGACTTCAA, R-TCTCTTCCTCTTGTGCTCTT; hCOL2A1 F-CAACCAGATTGAGAGCATCC, R-GGTCAATCCAGTAGTCTCCA; hACAN F-TCGAGGACAGCGAGGCC, R-TCGAGGGTGTAGCGTGTAGAGA; hBGLAP F-GAAGCCCAGCGGTGCA, R-CACTACCTCGCTGCCCTCC.

### 2.8 Statistical analysis

All error bars on data represent standard error from the mean. Statistical significance was determined at 95% confidence level by one-way analysis of variance (ANOVA), with pairwise comparisons conducted post hoc using the Tukey test.

## 3 Results

### 3.1 Substrate analysis

**Fig. 1:**
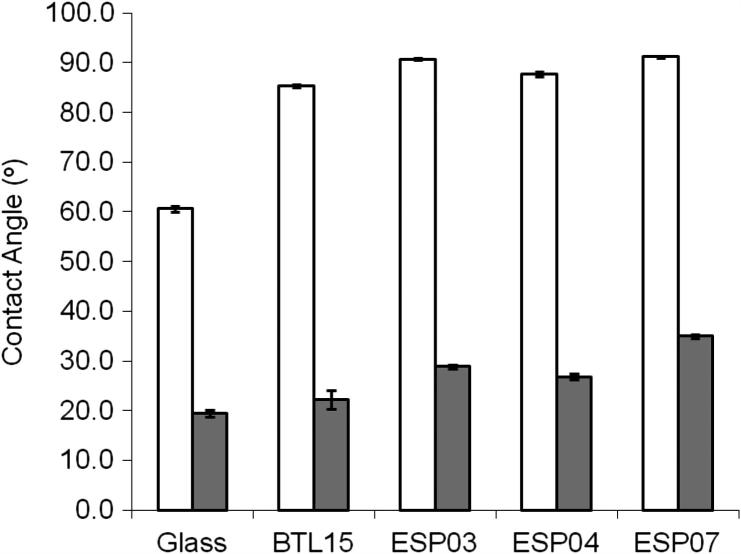
Dynamic contact angle measurements. All polyacrylates were significantly more hydrophobic than glass (*p* < 0.05, Tukey test) and most displayed significantly different hydrophilicity and hydrophobicity from each other (*p* < 0.05, Tukey test); advancing angle (white); receding angle (grey). The only combination with no significant differences between both advancing and receding angles was ESP03 with ESP07. Hysteresis, the difference between advancing and receding angles, was significantly higher than glass on all polyacylates (*p* < 0.05, Tukey test). Results represent the mean from a minimum of five replicates ± SEM.

**Fig. 2:**
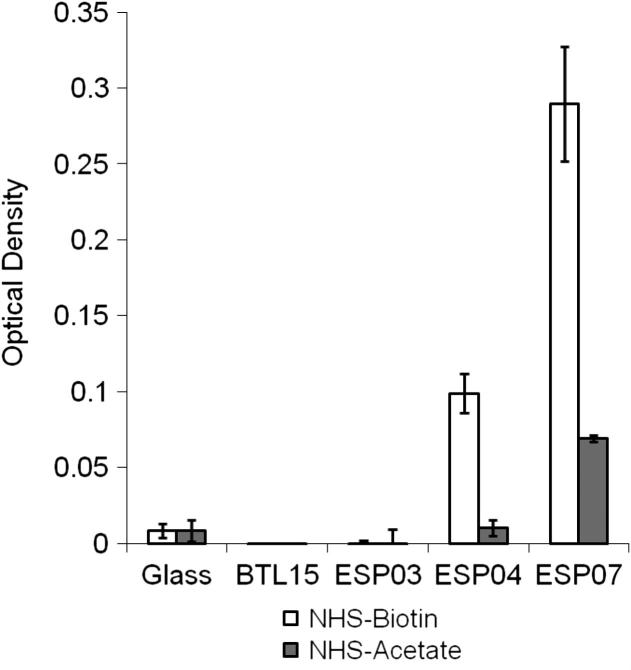
Surface amine assay. The availability of amine groups present at the surface of each substrate was quantified using NHS-biotin. Pre-treatment with NHS-acetate demonstrates significant knock-down of signals. Asterisk indicates statistically significant levels of surface amine compared to control (*p *< 0.05, Tukey test). Results represent the mean of three replicates ± SEM.

Physiochemical properties of the polyacrylate substrates are summarized in Table 1. DCA measurements demonstrated high hysteresis, suggesting distinct regions of hydrophobic and hydrophilic behaviour (Fig. 1). All substrates tested were significantly more hydrophobic than glass controls, with the higher amine content polyacrylates (ESP03, ESP04 and ESP07) having higher contact angles than BTL15. The trend from most hydrophilic to most hydrophobic was BTL15 > ESP04 > ESP03 > ESP07. Little difference in elemental composition was demonstrated by XPS analysis, which was close to theoretical values calculated from the cumulative individual monomeric unit contributions for C, N and O to the overall polymer chain (Table 2). High resolution C1s and N1s spectra demonstrated the polyacrylate surfaces were complex mixtures of hydroxyl, carboxyl and amine functional groups. The lack of sensitivity of XPS did not allow differences to be demonstrated quantitatively. As expected, amine content was lowest in BTL15 (0.6%) followed by ESP03 (1.4%) and highest in ESP04 (1.6%), and oxygen content was highest in BTL15 (21.1%) followed by ESP03 and ESP04 (20%). Interestingly, in ESP03 and ESP04, the proportion of oxygen was ∼ 3% higher at the surface (15°) than in the bulk (45°) and nitrogen was 0.1% lower at the surface than in the bulk, whereas no difference was observed in BTL15. ESP07 was not analysed by XPS, but theoretical values suggest similar element concentrations to ESP04. The NHS-biotin assay demonstrated significantly more surface amine on ESP07 (*p *< 0.05) than all other substrates, followed by ESP04 (*p *< 0.05); however, the assay was unable to detect any signal above background on BTL15 and ESP03 (Fig. 2).

### 3.2 mMSCs cultured on ESP04 undergo chondrogenesis

**Fig. 3:**
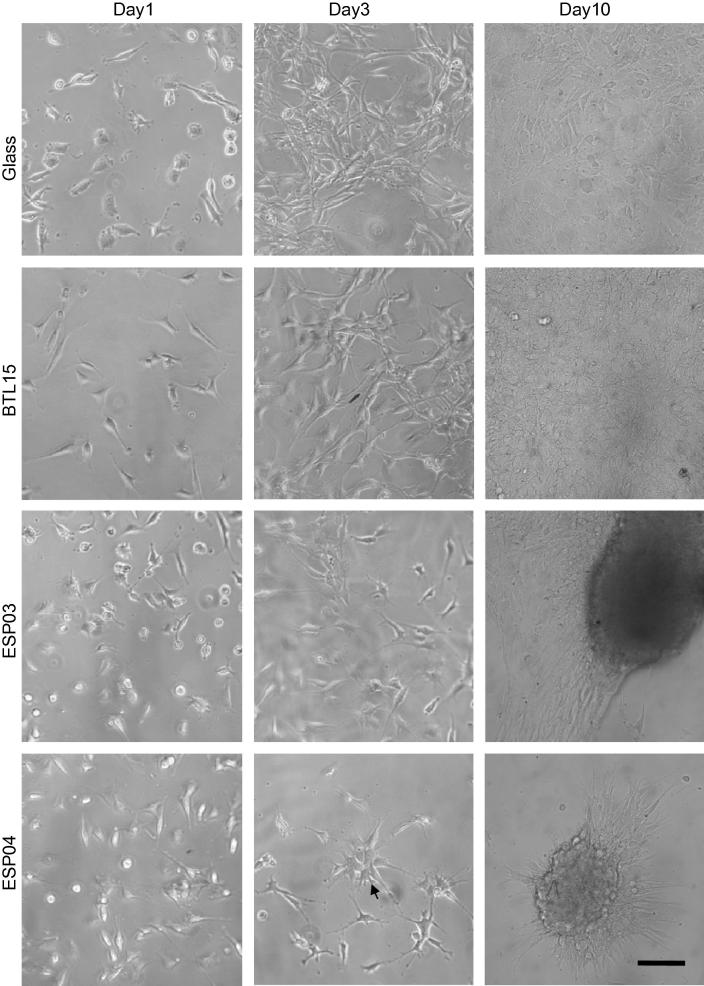
Typical mMSC behaviour on polyacrylate substrates. D1 mMSCs were seeded at 1 × 10^4^ cells per well, cultured for 10 days and observed in culture on plain glass, BTL15, ESP03 and ESP04. Representative images are shown of cells cultured for 1, 3 and 10 days. On glass and BTL15, cells attached and grew as a monolayer. Cells on ESP03 initially grew as a monolayer, but then compacted to form large aggregates distributed within the monolayer. On ESP04, cells formed multi-layered aggregates. Scale bar: 100 μm.

The effect of the polyacrylate substrates, BTL15, ESP03 and ESP04, on the morphology of mMSCs was tested in standard culture medium. Throughout the culture period, cells grown on glass and BTL15 substrates grew in monolayers and displayed the characteristic spindle-shaped morphology of mMSCs. In contrast, cells on ESP04 began to form mono-layered aggregates of ∼100 μm at day 3, and by day 10, numerous multi-layered aggregates of ∼200 μm were visible (Fig. 3). A few multi-layered aggregates were also present on ESP03 by day 10, but unlike those on ESP04, aggregates on ESP03 arose because cells at the edge of the monolayer detached from the substrate, and then retracted to form a multi-layer (Fig. S1).

**Fig. 4:**
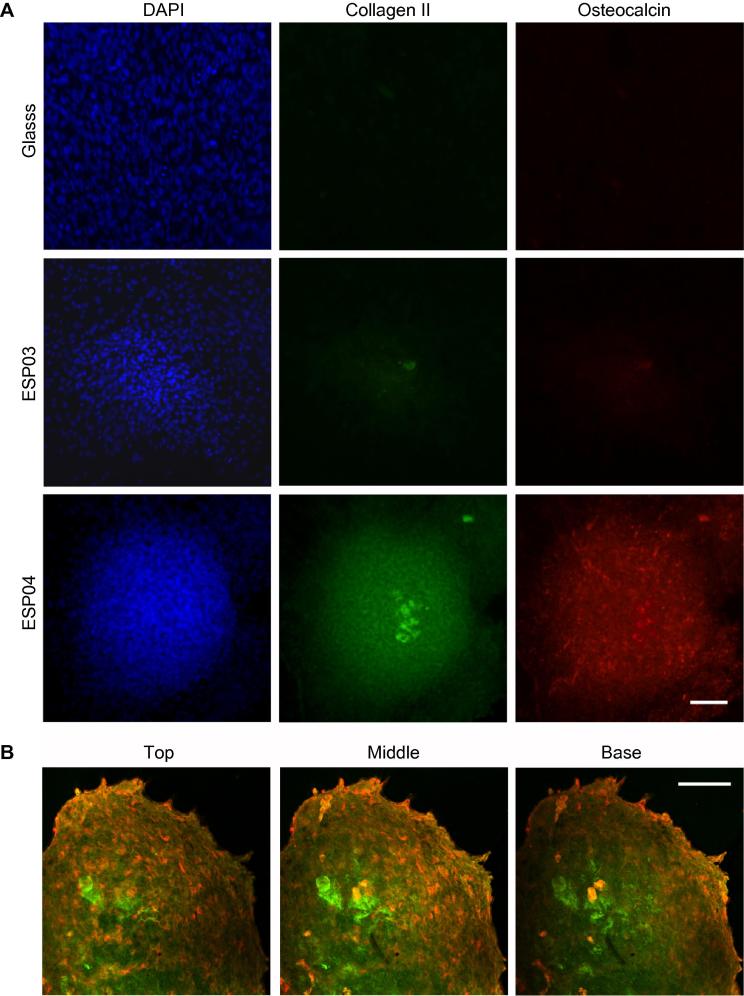
Chondrocyte and mineralization markers were detected in mMSCs cultured on polyacrylate substrates for 18 days; representative images are shown. (A) Cells were stained for collagen II (green) and osteocalcin (red), and nuclei were stained with DAPI (blue). Immunostaining for osteocalcin demonstrates increased expression throughout the aggregates whereas collagen II is typically localized to the centre of the aggregates. No staining was detectable in glass control substrates or BTL15 (not shown). Scale bar: 200 μm. (B) Confocal microscopy of 18 day aggregates confirms that collagen II is located at the centre of aggregates, and osteocalcin is at the periphery. Scale bar: 100 μm.

**Fig. 5:**
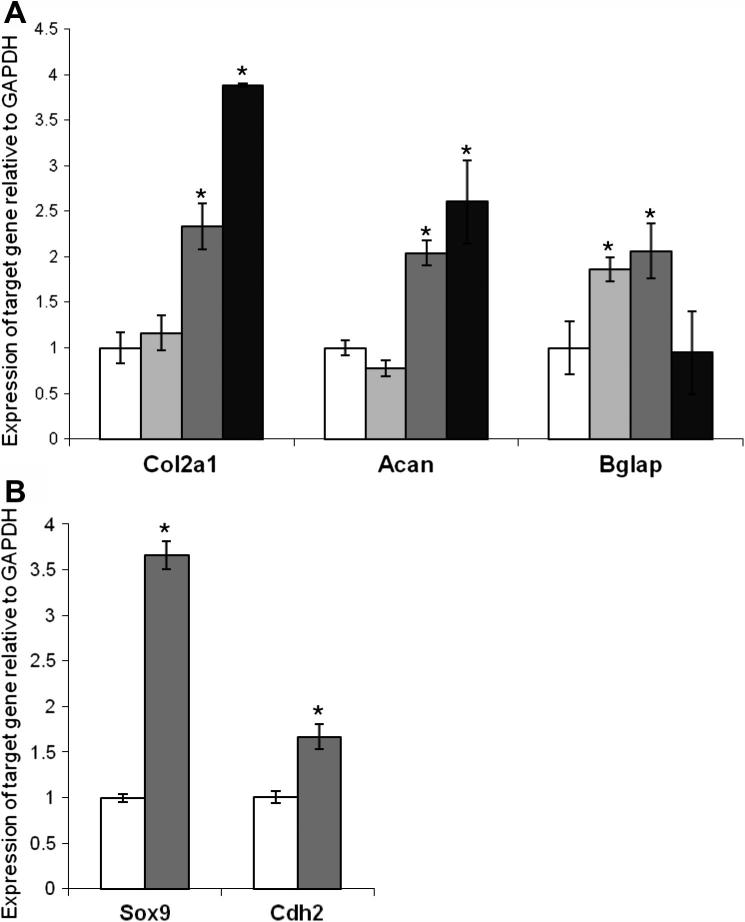
Up-regulation of chondrocyte markers in mMSCs cultured on ESP04. (A) Real time RT-PCR was conducted on mMSCs for the chondrocyte markers, *Col2a1* and *Acan*, and the mineralization marker, *Bglap* following 18 day culture on glass control (white) and polyacrylate substrates ESP03 (light grey) and ESP04 (dark grey). Expression levels in pellet cultures exposed to chondrogenic factors is included for comparison (black). (B) Real time RT-PCR was conducted on mMSCs for the early chondrocyte markers, *Sox9* and *Cdh2*, following a 2 day culture period on polyacrylate substrates. Data are normalized to expression on plain glass controls. The reference gene is *Gapdh*. Asterisk indicates data points which are significantly different from controls (*p *< 0.05, Tukey test). Results represent the mean of three biological replicates ± SEM.

Following an 18 day culture period, large aggregates were visible across ESP04 substrates, which typically ranged from 300 μm to 600 μm in diameter. Both collagen II and osteocalcin were observed within the aggregates on ESP04, but were barely detectable within aggregates on ESP03. No staining was detected in cells cultured on control (glass) or BTL15 (not shown) substrates over the same time period (Fig. 4A). Confocal microscopy of the immunostained mMSC aggregates on ESP04 showed that collagen II was mainly present at the centre of the aggregates, whereas osteocalcin was predominantly localized at the periphery (Fig. 4B). qPCR analysis at this time point showed that expression levels of *Col2a1* (collagen II-encoding gene) and *Acan* (aggrecan-encoding gene) increased 2.3- and 2-fold, respectively, in mMSCs cultured on ESP04 in comparison to negative controls, but the extent of up-regulation was less than that observed in pellet cultures (3.9- and 2.6-fold) for *Col2a1* and *Acan*, respectively (Fig. 5A). mMSCs cultured on ESP03 did not up-regulate *Col2a1* and *Acan*, suggesting that the cells were not differentiating into chondrocyte-like cells on this substrate. Expression levels of *Bglap* (osteocalcin-encoding gene) were significantly increased in mMSCs cultured on both ESP03 (1.9-fold) and ESP04 (2.1-fold) (Fig. 5A), in comparison to mMSCs on control substrates, whereas no increase was observed in pellet cultures. Expression levels of genes encoding the adipocyte markers, fatty acid binding protein (FABP) and peroxisome proliferator-activated receptor gamma (PPAR-γ) were not increased on the polyacrylate substrates in comparison to controls (results not shown). qPCR analysis of mMSCs that had been cultured for only 2 days on ESP04, a time point at which the cells have not yet begun to aggregate, showed that levels of the early chondrogenic markers, *Sox9* and *Cdh2* (N-cadherin-encoding gene), were 3.7- and 1.7-fold higher compared to mMSCs on control substrates (Fig. 5B), suggesting that chondrogenic induction of mMSCs on ESP04 occurs prior to aggregation.

### 3.3 KSCs cultured on ESP04 undergo chondrogenesis

**Fig. 6:**
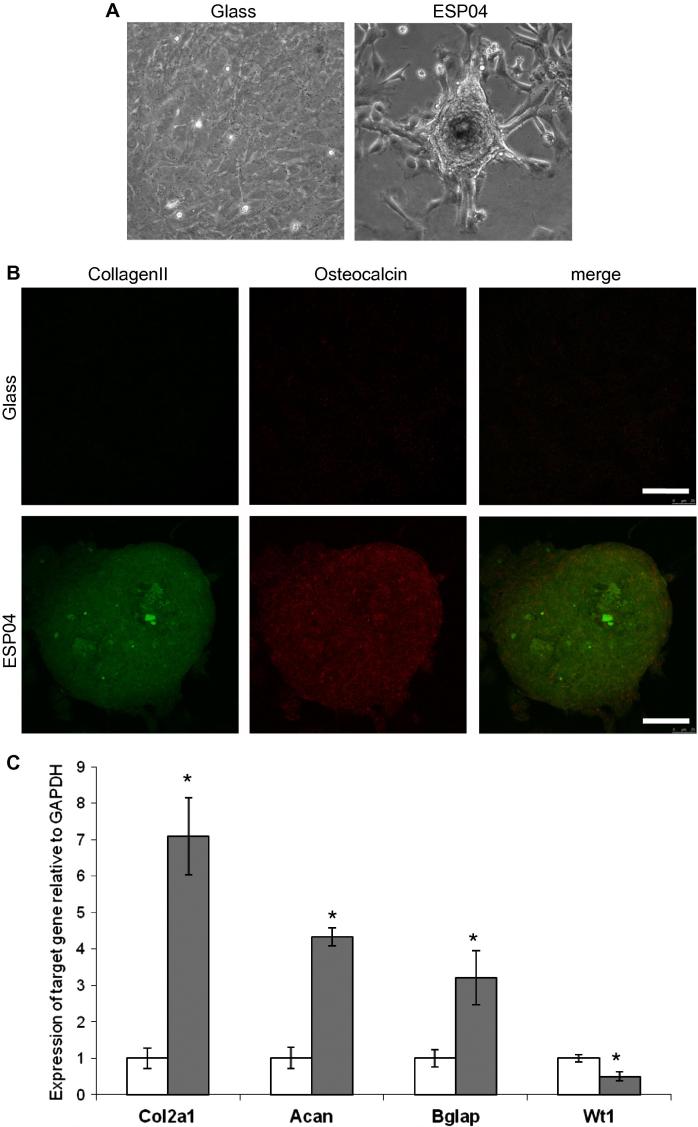
KSCs aggregate and express chondrocyte markers on ESP04. (A) KSCs were seeded at 1 × 10^4^ cells onto glass and ESP04 substrates. Representative images are shown following a 14 days culture period. (B) Cells were stained for collagen II (green) and osteocalcin (red), and nuclei were stained with DAPI (blue). Confocal image demonstrates the presence of collagen II throughout the aggregates, and osteocalcin localized to the periphery. Scale bar: 50 μm. (C) Real time RT-PCR was conducted on KSCs for the chondrocyte markers, *Col2a1* and *Acan*, the mineralization marker, *Bglap*, and the kidney progenitor marker, *Wt1*, following a 14 day culture period on glass (white) and ESP04 (grey) substrates. Data are normalized to expression on plain glass controls. The reference gene is *Gapdh*. Asterisk indicates data points which are significantly different from controls (*p *< 0.05, Tukey test). Results represent the mean of three biological replicates ± SEM.

The chondroinductive properties of ESP04 were further analysed with mouse KSCs which, like MSCs, are capable of undergoing adipo- and osteogenesis [33]. Due to the fact that BTL15 and ESP03 were unable to induce mMSCs to undergo chondrogenesis (Figs. 4 and 5), these substrates were not tested on KSCs. Following 14 days of culture on ESP04, KSCs had formed multi-layered aggregates (Fig. 6A), in similar numbers to mMSC aggregates and typically ∼200 μm in diameter. Confocal microscopy showed that cells within the aggregates expressed collagen II and, similarly to mMSCs, some KSCs at the periphery tended to express osteocalcin (Fig. 6B). qPCR showed that the expression levels of *Col2a1*, *Acan* and *Bglap* were increased 7.1-, 4.3- and 3.2-fold, respectively, in KSCs cultured on ESP04 in comparison to cells cultured on glass substrates, confirming that the cells were progressing down a chondrocyte lineage. Furthermore, expression levels of the KSC marker, *Wt1*, were decreased by 0.5-fold in KSCs cultured on ESP04 substrates, indicating loss of the KSC phenotype (Fig. 6C).

### 3.4 hMSC chondrogenesis on polyacrylate substrates

**Fig. 7:**
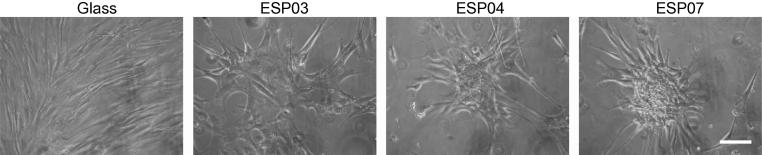
hMSC behaviour on polyacrylate substrates. hMSCs were seeded at 5 × 10^3^ cells per well onto plain glass, ESP03, ESP04 and ESP07 substrates. Representative images are shown of cells cultured for 7 days. Cells grew as evenly distributed monolayers on glass. On ESP03, ESP04 and ESP07, cells formed aggregates; however, multi-layering was only observed on ESP07. Scale bar: 200 μm.

**Fig. 8:**
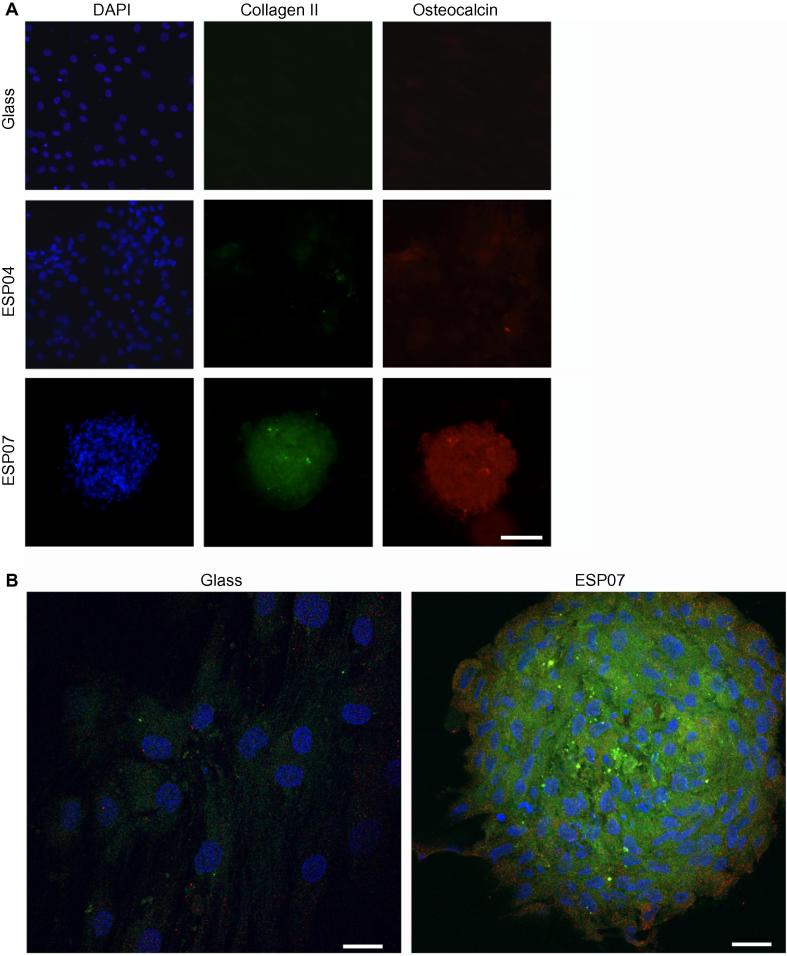
Chondrocyte and mineralization markers were detected in hMSCs cultured on polyacrylate substrates. Representative images are shown following a 20 day culture period. (A) hMSCs on glass, ESP04 and ESP07, were stained for collagen II (green) and osteocalcin (red), and nuclei were stained with DAPI (blue); scale bar: 200 μm. (B) Confocal microscopy of hMSCs cultured on glass and ESP07 confirms that while only background staining is observed in cells cultured for 20 days on glass, staining for collagen II is present throughout the hMSC aggregates that formed on ESP07. Weak staining for osteocalcin is present at the periphery of aggregates on ESP07. Scale bar: 50 μm.

The effect of the polyacrylate substrates on the morphology of hMSCs cultured in standard medium (without growth factors) was investigated. A polyacrylate with further enhanced amine content (ESP07) was added to the investigation at this point. By day 7, aggregates had formed on both ESP04 and ESP07, but whilst only monolayered aggregates were present on ESP04, compact, multi-layered aggregates were present on ESP07 (Fig. 7), in similar numbers to mMSC aggregates and typically ∼500 μm in diameter. BTL15 and ESP03 were omitted from further experiments due to their inability to induce the hMSCs to aggregate. Immunofluorescence analysis following a 20 day culture period showed that collagen II and osteocalcin were expressed within the multi-layered hMSC aggregates on ESP07, but were barely detectable on ESP04 (Fig. 8A). Confocal microscopy of the immunostained hMSC aggregates on ESP07 showed that collagen II was mainly present at the centre of the aggregates, whereas osteocalcin was predominantly localized at the periphery (Fig. 8). Thus, it appeared that ESP07 (but not ESP04) could induce hMSCs to differentiate down the chondrocytic lineage in the absence of exogenous growth factors. The size of the hMSC aggregates following a 20 day culture period were ∼500–1000 μm (Fig. 8B).

**Fig. 9:**
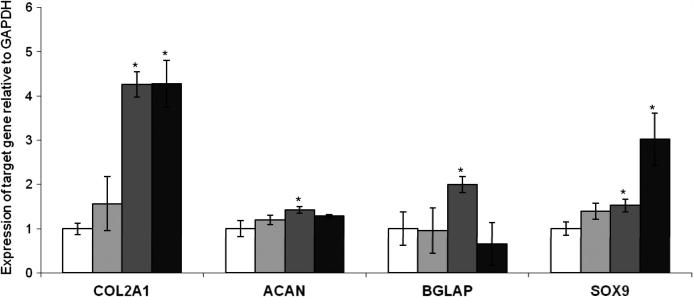
Up-regulation of chondrocyte markers in hMSCs cultured on ESP07. Real time RT-PCR was conducted on mMSCs for the chondrocyte markers, *COL2A1*, *ACAN* and SOX9, and the mineralization marker, *BGLAP,* following a 20 day culture period on glass control (white) and polyacrylate substrates ESP04 (light grey) and ESP07 (dark grey). Data are normalized to expression on plain glass controls. The reference gene is *GAPDH*. Expression levels in pellet cultures exposed to chondrogenic factors is included for comparison (black). Asterisk indicates data points which are significantly different from controls (*p *< 0.05, Tukey test). Results represent the mean of three biological replicates ± SEM.

Following a 20 day culture period, there was a significant up-regulation of *SOX9*, *BGLAP*, *COL2A1* and *ACAN* in hMSCs cultured on ESP07 (Fig. 9) in comparison with glass and ESP04. The expression levels of *COL2A1* in hMSCs cultured on ESP07 were comparable to those of hMSCs under standard pellet culture conditions (i.e., in the presence of exogenous growth factors), approximately 4.3-fold higher than plain glass controls. Interestingly, the expression levels of *ACAN* were marginally higher (1.1-fold), and those of *SOX9* lower (0.5-fold), in hMSCs cultured on ESP07 compared to those in pellet culture, suggesting that maturation of chondrocyte-like cells might be accelerated on ESP07 (Fig. 9).

## 4 Discussion

Using novel polyacrylate substrates, generated by free radical polymerization with proprietary controlled process techniques, we have investigated the effect of surface chemistry on the chondrogenic potential of different mesenchymal cell types. Our study has shown that by controlling the composition and distribution of surface functional groups, we can direct the differentiation of mMSCs, hMSCs and mouse KSCs down a chondrocyte lineage. To our knowledge this is the first study to demonstrate chondrogenic induction via surface chemistry, without the requirement for exogenous growth factors such as TGF-βs.

To design the novel polyacrylic surfaces we used the theoretical structural and functional chemistry of peptide sequences as a design template [36–39]. This allowed us to choose the appropriate acrylic monomer units, each carrying one or more component functional group chemistries, which, once synthesized in the appropriate compositional ratios, would create the target mimetic surface. Neutral ”spacer” monomeric units were used to create the spatial separation of functional group chemistries. Bulky pendant side groups were used to control and modify the degree of chain rotation and therefore the contextual presentation of surface chemistries.

An interesting finding of our study is that subtle changes in the chemical composition of substrates can lead to dramatic differences in cell behaviour. For instance, whilst XPS analysis showed no significant differences in the elemental composition of any of the polyacrylate substrates, chondrogenesis inducing ability was strikingly different, with BTL15 and ESP03 being ineffective, and ESP04 and ESP07 being chondroinductive. It should not be surprising that XPS was unable to distinguish between the surfaces, as the expected differences in elemental composition are small, but the fact that the cellular response varied demonstrates the very significant importance of the potential structural and spatial variation of the chemical functionalities provided by the combination of monomer composition and spacer units. DCA analysis indicated that surface wettability appeared to have no effect on chondrogenesis. However, there did appear to be a positive correlation between chondroinductive ability and surface amine content, as only the chondroinductive substrates, ESP04 and ESP07, displayed a significantly higher surface amine content than controls. Indeed, surface amine availability appears to be a key factor in the chondrogenic capacity in this range of polyacrylate substrates, with a clear enhancement of chondroinduction from ESP03 to ESP04, in mMSCs, and ESP04 to ESP07, in hMSCs, with increasing surface amine. This result is consistent with previous reports indicating that substrates modified with amine functional groups can promote MSC chondrogenesis [22,23], though it is important to note that in contrast to the polyacrylates used in our own work, the substrates used in these earlier studies were not sufficient to induce chondrogenesis in the absence of TGF-β supplementation [22,23]. Taken together with our own findings, these results indicate that a particular combination of chemical functionalities is required to induce chondrogenesis, rather than just amines alone, where other features of the polymers may interact with amine groups to construct the chondroinductive surface. A recent study by Park et al. has shown that substrate stiffness can also regulate MSC chondrogenesis; in this case, soft substrates induced chondrogenesis, whereas stiff substrates did not [29]. However, Park et al. reported that in the absence of TGF-β supplementation, MSCs on soft substrates did not differentiate exclusively to chondrocytes, but also differentiated to adipocytes, generating a heterogeneous population. In contrast, the ESP04 and ESP07 polyacrylate substrates used in the current study were able to direct the differentiation of mMSCs and hMSCs, respectively, to a chondrocyte-like phenotype, without any evidence of adipocyte differentiation.

A key question arising from our study is how can the polyacrylate substrates induce chondrogenesis in the absence of TGF-βs? Members of the TGF-β superfamily tend to be secreted from cells as proproteins, comprising a mature domain that binds to TGF-β cell surface receptors, and a prodomain that tethers the proprotein to the ECM, thereby creating a high local concentration in the ECM surrounding the TGF-β-secreting cells [40]. It should be noted that MSCs do in fact express their own TGF-βs [41,42]; indeed,TGF-βs can reach fairly high concentrations in the supernatant of MSCs cultured in vitro [42]. Thus, it is possible that TGF-β supplementation is not required to induce MSC chondrogenesis on the polyacrylate substrates used here, because the TGF-βs secreted from the MSCs are able to bind to these substrates, creating a high local concentration that is sufficient to induce signalling. Alternatively, direct cell–substrate interaction could activate integrins, which can in turn potentiate TGF-β signalling by activating latent TGF-βs, or by interacting with TGF-β receptors [43].

During limb bud development, TGF-β signalling in mesenchymal cells induces expression of the genes encoding fibronectin, Sox9 and N-cadherin, which are responsible for driving the subsequent aggregation of the mesenchymal cells [7,9,10]. However, if MSCs are cultured in vitro on control substrates, TGF-β supplementation is not sufficient to induce chondrogenesis and the cells need to be grown in three-dimensional (3-D) pellet or micromass cultures [18]. Thus, in our study, we wished to establish if the cellular aggregation observed on the chondroinductive substrates was a prerequisite for chondrogenesis or, alternatively, occurred as a consequence of chondrogenic induction, as previously shown [44]. Importantly, we found that *Sox9* and *Cdh2* were induced in MSCs cultured on the ESP04 substrate before the cells had started to aggregate, showing that aggregation was occurring secondary to chondrogenic induction, similarly to the sequence of events that occurs during limb bud chondrogenesis [4]. These results are consistent with previous studies showing that if MSCs are cultured in scaffolds or hydrogels that prevent aggregation, chondrogenesis can still proceed, suggesting that with the appropriate artificial environment, aggregate formation may not be necessary to induce chondrogenesis [25,27]. Whilst chondrogenesis may be initiated by polyacrylate substrates, aggregation and condensation of the MSCs appear to be required for the progression of chondrogenesis on these polyacrylates. This is demonstrated by the lack of chondrocyte markers detected in hMSCs cultured on ESP04 which, while aggregating and expressing SOX9, did not condense and form a multi-layer. The subsequent aggregation and condensation of the MSCs likely drives further progression down a chondrocyte lineage. Indeed cells within the 3-D aggregates will no longer be in direct contact with the substrates and will therefore be reliant upon paracrine signalling and cell–cell interactions.

A second question arising from our study is why do MSCs cultured on the chondroinductive polyacrylate substrates form aggregates, whereas MSCs cultured on control substrates do not, even in the presence of TGF-β? (results not shown). Previous studies have attributed aggregation on amine surfaces to reduced cell adhesion or altered protein adhesion [22,24,45]; however, in our study, the expression pattern of chondrocyte genes suggests direct chondrogenic induction prior to aggregation. Studies have also shown that biomaterials functionalized with RGD peptides can promote MSC chondrogenesis in vitro [25–27], most likely because MSC interaction with the RGD peptides mimics the interaction that occurs between mesenchymal cells and the RGD motif of fibronectin in the developing limb bud. Our results show that the chondroinductive polyacrylates were not only able to induce chondrogenesis, but were also able to support subsequent aggregation. We suggest that the ability of the cells to aggregate following chondrogenic induction on these substrates could be due to two possible scenarios: either the MSCs directly interact with the substrates, and it is this interaction that promotes their aggregation, or alternatively, the MSCs interact with ECM molecules that are bound to the polyacrylate substrates in conformations that induce cell aggregation.

## 5 Conclusions

In summary, our results show that novel biomimetic polyacrylate substrates can direct mMSCs, hMSCs and mouse KSCs to undergo chondrogenesis in the absence of exogenous TGF-βs. Our study thus highlights the importance of surface group functionality and distribution in regulating stem cell behaviour. The process of chondrogenesis on the polyacrylate substrates appeared similar to that which occurs in standard pellet cultures. However, a key advantage of the chondroinductive polyacrylate substrates is that there is no requirement for supplementation with expensive growth factors, thus facilitating scale-up and simplifying the regulatory pathway were the technique to be incorporated into a therapeutic application.

## Figures and Tables

**Table 1:** Physiochemical properties of polyacrylate substrates.^a^

Property	BTL15	ESP03	ESP04	ESPO7
Amine content	+	++	+++	+++
Carboxylic acid content	++	++	+	−
Hydroxyl content	++	−	−	+
Wettability				
Advancing angle	85.3 ±0.3	90.8 ±0.2	87.7 ±0.3	91.2 ±0.2
Receding angle	22.2 ±1.8	28.9 ±0.4	26.8 ±0.6	35.0 ±0.3
Hysteresis	63.1 ±1.5	61.9 ±0.4	60.9 ±0.4	56.2 ±0.4
Glass transition temperature /°C	31.4	32.8	34.7	36.7

a +, ++, +++ indicates relative content of the different groups (i.e., NH_2_, COOH, OH).

**Table 2:** XPS elemental analysis and theoretical elemental composition of polyacrylate substrate surfaces.

Substrate	Angle^a^	C	N	O
BTL15	45	77.80	0.60	21.60
BTL15	15	78.30	0.60	21.10
BTL15	Theoretical^b^	74.14	1.06	24.79
ESP03	45	81.99	1.48	16.76
ESP03	15	78.55	1.32	19.98
ESP03	Theoretical	74.88	1.64	23.47
ESP04	45	80.74	1.67	17.34
ESP04	15	77.12	1.57	20.41
ESP04	Theoretical	75.11	2.05	22.83
ESP07	Theoretical	74.73	2.18	23.09

a Each polymer was examined at 45° and 15° angles to the surface corresponding to 6 and 2 nm depths. Proportions of carbon, oxygen and nitrogen were calculated by curve fitting analysis of survey spectra and displayed as a percentage of totals.

b Theoretical values were provided by Biomer Technology Ltd.

## Appendix A Figures with essential colour discrimination

Certain figures in this article, particularly Figures 4, 6 and 8, are difficult to interpret in black and white. The full colour images can be found in the on-line version, at http://dx.doi.org/10.1016/j.actbio.2012.12.007).

## Appendix A Supplementary data

Supplementary data 1 Typical aggregate formation on ESP03 substrates. D1 mMSCs were seeded at 1 × 10^4^ per well and imaged following a 6 day culture period. Cells at the edge of the monolayer detached from the substrate, and then retracted to form a multi-layer. Scale bar: 100 μm.
