# Supplementary figures and images for: Induction of mesenchymal stem cell chondrogenesis by polyacrylate substrates
Source: Acta Biomater. 2013 Apr;9(4):6041–51. doi: 10.1016/j.actbio.2012.12.007 (PMC3594746; doi:10.1016/j.actbio.2012.12.007)

## Slide 1
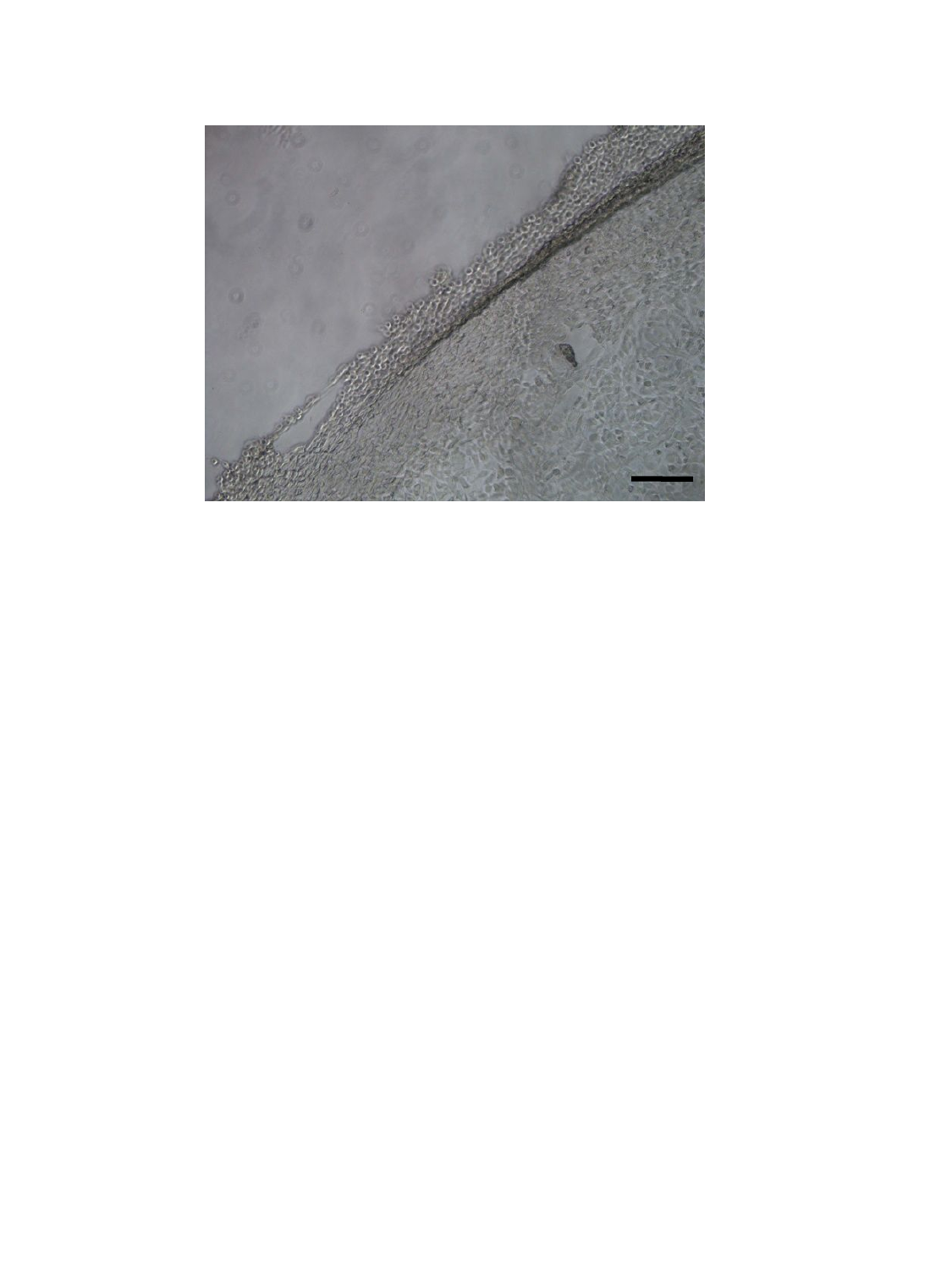

Supplement: Supplementary data 1 — Typical aggregate formation on ESP03 substrates. D1 mMSCs were seeded at 1 × 10^4^ per well and imaged following a 6 day culture period. Cells at the edge of the monolayer detached from the substrate, and then retracted to form a multi-layer. Scale bar: 100 μm. [file mmc1.ppt]
